# Endovascular management of a spontaneous aorto-caval fistula resulting from abdominal aortic aneurysm: case report and literature review

**DOI:** 10.3389/fcvm.2025.1518025

**Published:** 2025-01-30

**Authors:** Dongbo Wu, Cheng Peng, Zhiyong Zhou, Jie Chen, Bin He, Zhidong Ye

**Affiliations:** ^1^ Department of Vascular Surgery, Xing'an League People’s Hospital, Ulanhot, China; ^2^ Department of Cardiovascular Surgery, China-Japan Friendship Hospital, Beijing, China

**Keywords:** aorto-caval fistula, abdominal aortic aneurysm, endovascular stent-graft repair, Endoleaks, case report

## Abstract

**Background:**

Aorto-caval fistula is a rare complication of abdominal aortic aneurysms that can occur spontaneously, iatrogenically, or traumatically, associated with high morbidity and mortality. Endovascular stent graft repair represents a practical approach to managing this fatal condition.

**Case presentation:**

A 75-year-old male patient was admitted to the nephrology department of our hospital, complaining of acute back pain, hematuria, and repeated vomiting for one week. The laboratory assessments yielded high creatinine levels, indicating an acute renal impairment. Computed tomography angiography revealed an aorto-caval fistula complicating infrarenal abdominal aortic aneurysm. The patient was successfully treated with the endovascular approach by deploying covered stent grafts that completely excluded the fistula.

**Conclusion:**

Utilizing covered stent grafts for endovascular management of aorto-caval fistula is a good alternative to conventional surgery, especially in elderly high-risk patients.

## 1 Introduction

Primary aorto-caval fistula (ACF) is a devastating and rare complication of abdominal aortic aneurysms (AAA), accounting for 0.2%–1.3% of all aneurysms and in around 3% of ruptured AAA (1). The traditional management of ACF secondary to AAA was an open surgical procedure for a long time. However, due to excessive intraoperative blood loss, pulmonary embolism, and cardiac decompensation, the retrospective assessments of perioperative mortality for this condition could be up to 60% (2). With the development of the aortic-covered stent-graft technique, endovascular repair provides a minimally invasive alternative in managing ACF (3). Herein, we present a case involving an old male patient who developed a spontaneous ACF resulting from AAA. The fistula was effectively treated by using a covered stent graft.

## 2 Case report

### 2.1 Case description

A 75-year-old male patient presented to our hospital with acute low back pain, hematuria, and repeated vomiting for one week. Physical examination revealed blood pressure of 115/75 mmHg, heart rate of 86 beats per minute, jugular distension, and pitting edema of bilateral lower limbs. Abdominal examination showed a pulsating mass and a continuous audible bruit over the abdomen on auscultation, suggesting an AAA. There was no shortness of breath. Laboratory findings noted renal impairment with a serum creatinine level of 5.57 mg/dl and anemia with a hemoglobin of 10.5 g/L.

### 2.2 Diagnostic assessment

**Figure 1:**
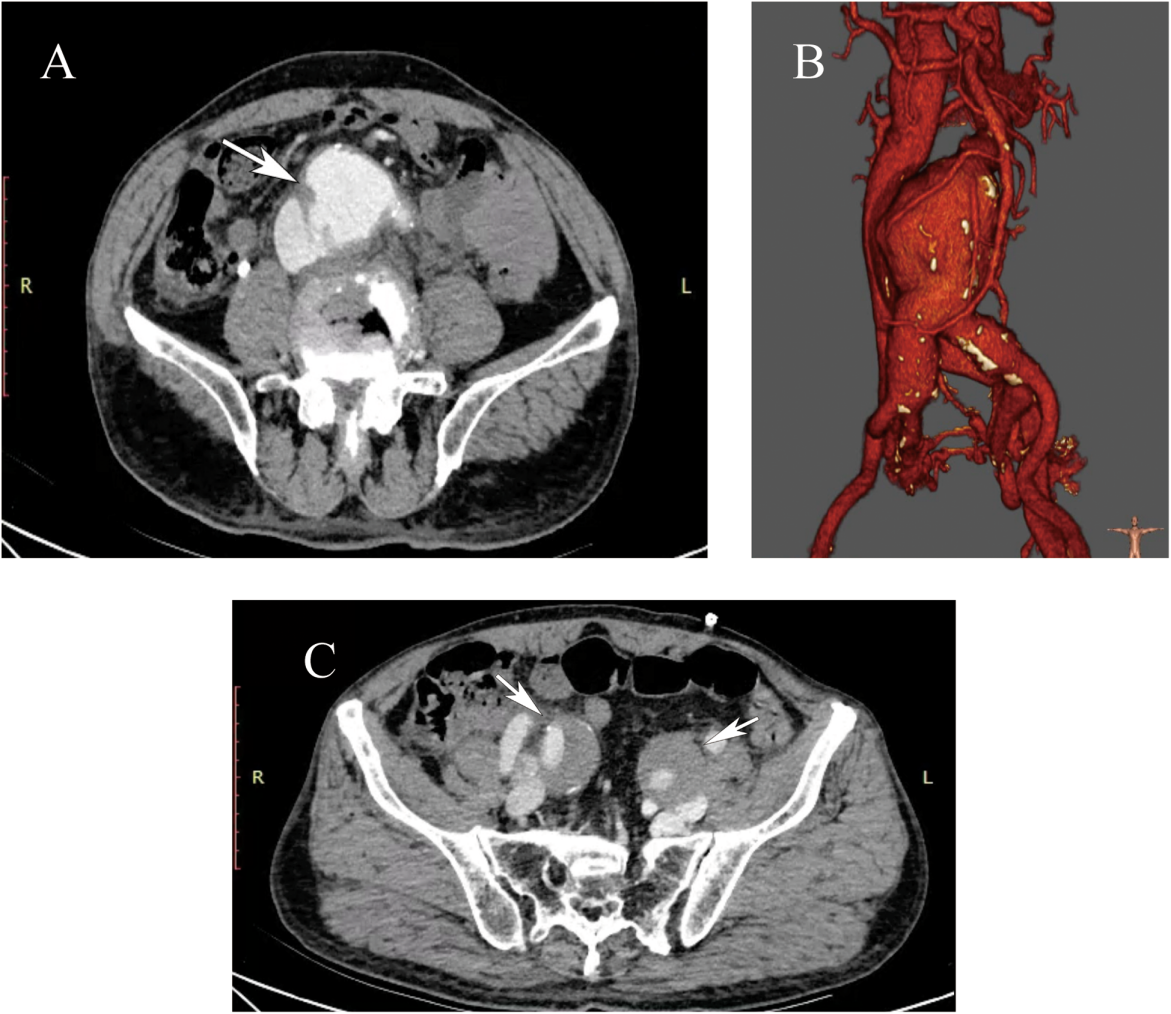
**(A)** The preoperative computed tomography angiography (CTA) showed a large abdominal aortic aneurysm (AAA) with associated aortocaval fistula (ACF, white arrowheads). **(B)** Three-dimensional reconstruction CTA showed an angulated aneurysm and tortuous aorto-iliac access. The maximum diameter of the AAA was approximately 8.7 cm. **(C)** The preoperative CTA showed bilateral internal iliac artery aneurysms (white arrowheads). The maximum diameter of the left internal iliac artery is approximately 32 mm, and the maximum diameter of the right internal iliac artery is approximately 34 mm.

The patient received the abdominal aorta ultrasound at first, which indicated aneurysmal dilatation of the abdominal aorta above the umbilical level, measuring approximately 8.6 × 7.0 cm. CDFI (Color Doppler Flow Imaging) indicated that an arterial spectrum is detectable within the inferior vena cava, with a blood flow velocity of approximately 86 cm/s. Based on the result of ultrasound, we further performed the CTA (computed tomographic angiography) scan for the patient, which demonstrated a large infrarenal AAA with a maximum diameter of 8.7 cm. During the arterial phase, the rapid contrast early filling of the inferior vena cava vein could be found, confirming the diagnosis of ACF (Figure 1A). There was one channel of contrast and no evidence of rupture. Angulated aneurysm and tortuous aorto-iliac access were also shown (Figure 1B). An echocardiogram revealed that the left ventricular ejection fraction (LVEF) was 65%. Since admission, we repeatedly performed complete blood counts (CBC), and the patient's WBC levels ranged from 4 to 5 × 10^9^/L, which is within the normal range of 3.5–9.5 × 10^9^/L. Additionally, the patient's body temperature remained normal throughout.

### 2.3 Treatment

Due to the large diameter of the aneurysm and the considerable size of the ACF, the patient was offered and elected to receive endovascular repair. Preoperative accurate measurements showed that the aneurysm neck angle is 44.4 degrees, the neck length is 52 mm, the proximal neck diameter is 25.5 mm, the distal neck diameter is 24.8 mm, the diameter of the left common iliac artery is 20.5 mm, the diameter of the right common iliac artery is 26.4 mm, the diameter of the left internal iliac artery is approximately 32 mm, and the diameter of the right internal iliac artery is approximately 34 mm (Figure 1C).

The operation was done successfully under general anesthesia. Bilateral femoral arteries were punctured using the Seldinger technique. After pre-positioning two Perclose ProGlide (Abbott), the 8F catheter sheath was inserted bilaterally. Heparin 5,000 U was administered. Due to the preoperative CTA showing bilateral internal iliac arteries diameters are greater than 30 mm, combined with considerations of the patient's advanced age and poor financial situation, we performed bilateral internal iliac artery embolizations for this patient first.

A guidewire and adjustable catheter were introduced into the right femoral artery and super-selectively advanced into the right internal iliac artery. Interlock detachable coils (Boston Scientific, USA) measuring 10 mm × 40 cm (2 pieces), 15 mm × 40 cm (1 piece), and 20 mm × 40 cm (1 piece) were deployed. Using the same method, we embolized the left internal iliac artery, deploying Interlock detachable coils (Boston Scientific, USA) measuring 15 mm × 40 cm (2 pieces) and 8 mm × 40 cm (1 piece). Angiography showed good embolization of bilateral internal iliac arteries.

**Figure 2:**
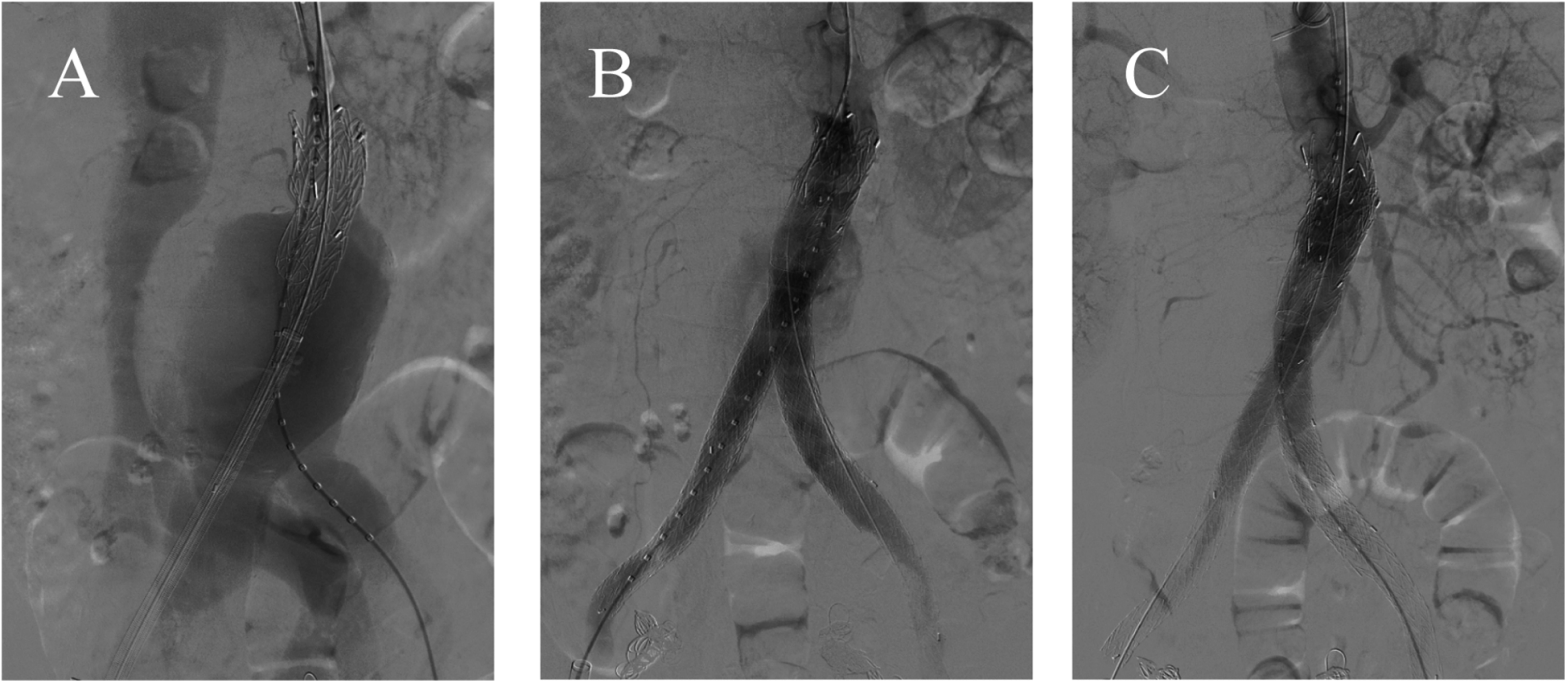
**(A)** The angiogram confirmed the large AAA with ACF. **(B)** After standard EVAR, intraoperative angiography showed the type Ia endoleak. **(C)** There was no endoleak after implanting an Excluder aortic stent-graft cuff.

Through the right femoral artery sheath, a guidewire and pigtail catheter were introduced. Initial angiography (contrast agent total 25 ml, 20 ml/sec, pressure 60 mmHg) revealed the formation of AAA and the diagnosis of ACF (Figure 2A). The central venous pressure was 30 mmHg. Measurements showed the normal abdominal aortic diameter to be approximately 2.7 cm. Based on the measurements, the abdominal aortic endograft system (W.L. Gore & Associates, Flagstaff, AZ) was selected, including a main body stent graft (28.5 mm × 14.5 mm × 14 cm), a left iliac branch (16 mm × 14.5 mm × 14 cm and 16 mm × 12 mm × 14 cm), and a right iliac branch (16 mm × 14.5 mm × 14 cm). The 8F sheath was exchanged for an 18F vascular sheath in the right common femoral artery, and a 16F vascular sheath was exchanged in the left common iliac artery. The super-slippy guidewire was exchanged for a stiff guidewire (Lunderquist), and the stent delivery system was introduced. Repeated angiography confirmed the position of the endograft, which was deployed.

Through the left femoral artery, a guidewire and catheter were introduced, but various catheters were unable to pass smoothly through the left abdominal aortic branch stent. The right brachial artery was punctured, and a 5F vascular sheath was placed. A guidewire and a 4F MPA catheter were used to super-selectively navigate into the left iliac branch. The guidewire was passed into the left femoral artery sheath and extracted externally, establishing a working pathway. A stiff guidewire was advanced into the left iliac branch, and after angiographic confirmation, the left and right iliac branch delivery systems were introduced and deployed. After standard EVAR, however, repeat angiography showed persistent type Ia endoleak (Figure 2B). The proximal landing zone was ballooned again. However, the endoleak was still existing. We finally treated it by implanting an 28.5 mm × 3.3 cm Excluder aortic stent-graft cuff (W.L. Gore & Associates, Flagstaff, AZ) (Figure 2C). At this time, the central venous pressure dropped to 10 mmHg.

### 2.4 Follow-up

**Figure 3:**
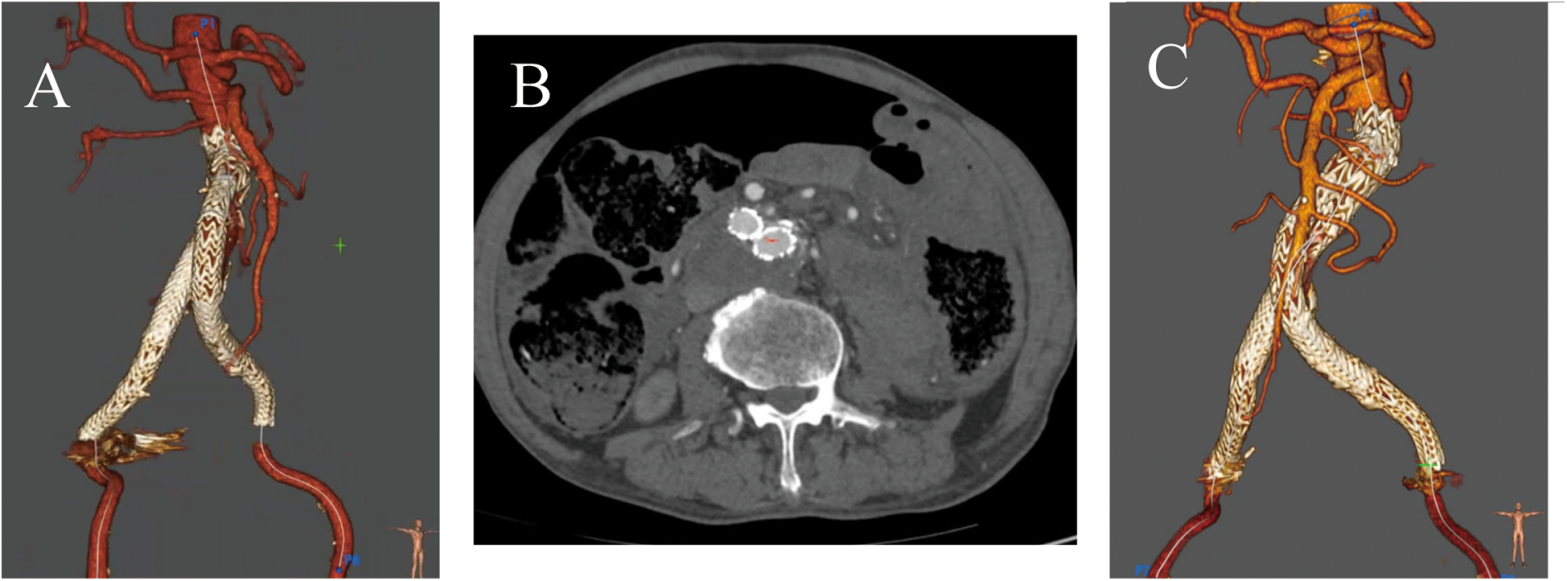
**(A)** Postoperative CTA showed no evidence of the fistula or the endoleaks. **(B,C)** At the 9-month follow-up, CTA showed no evidence of endoleak or fistula.

The post-operative CTA revealed the disappearance of leakage from the abdominal aorta to the inferior vena cava and no evidence of the endoleak (Figure 3A). The contrast agent used during the operation was around 120 ml. The patient did not undergo temporary hemodialysis after EVAR operation, since his urine output was normal. Approximately one week after the operation, his renal function and the edema of bilateral lower limbs improved satisfactorily. So, the patient was discharged uneventfully. The patient was followed up for nine months and remained in good condition. The patient did not experience buttock, ischemia or claudication after the surgery. CTA at the 9-month postoperative follow-up showed that the ACF had permanently vanished, with no endoleak observed (Figures 3B,C).

## 3 Discussion

There are several severe complications of AAA, including rupture, infection, aorto-enteric fistula, and aorto-caval fistulas (ACF). Among them, ACF is a rare and fatal complication in which AAA erodes or ruptures into the inferior vena cava (IVC). Most patients with ACF do not exhibit rupture syndrome of AAA upon admission, such as severe abdominal pain accompanied by hemorrhagic shock. In ACF patients, the symptoms of massive blood loss are usually presented as rapid arteriovenous shunt and secondary venous hypertension, including high output cardiac failure, oliguria, hematuria, bilateral pedal edema, and renal insufficiency (4). Due to the significant variation in clinical manifestations of ACF, it is usually hard to diagnose. In our case, the patient presented with back pain and hematuria. So, he was admitted to the nephrology department at first. After demonstrating AAA with ACF by CTA scan, the patient was transferred to our department and received surgery.

The first traumatic ACF case was described in 1831 by Syme et al., while the first successful surgical repair of ACF was reported in 1954 by DeBakey et al. (5, 6). Open surgical repair of ACF was direct suturing of the fistula from within the aneurismal sac, which was associated with high mortality and morbidity. Early surgical mortality is as high as 21%–51% based on the literature (7, 8). To reduce the risks of open surgery caused by the challenging dissection and massive bleeding, the first endovascular repair of ACF was first reported in 1999 (9). because of the minimally invasive advantage and less blood loss, endovascular stent-graft repair has soon become an evolving treatment option for ACF in the last 20 years.

However, endovascular repair for ACF has its complications. The studies found that ACF patients with hostile neck anatomy developed perioperative type I endoleak more frequently, such as a great angle at the proximal neck or a short proximal neck. As in our case, the Type Ia endoleak during EVAR can be attributed to a highly angled neck, measured at 44 degrees, which made it challenging for the stent graft to properly align and form a secure seal against the vessel wall. This misalignment can create gaps, leading to an endoleak, and we addressed this issue by using proximal cuff extensions. Moreover, persistent communication between the aneurysm sac and IVC can, theoretically, lead to a type II endoleak. Some cases presenting the type II endoleaks after endovascular stent-grafting of AAA with ACF were reported. In addition, a small number of cases develop Type III endoleak postoperatively. To further our understanding of the management of Endoleaks After endovascular stent-graft of AAA With ACF, we conducted searches of AAA, endovascular treatment, ACF, and endoleak in the medical databases. There have been around 20 clinical cases described (Table 1) (3, 10–21). Among them, 5 cases were Type I endoleak, 14 cases were Type II endoleak, and 1 case was Type III endoleak. The primary ACF fistula site was at the aortocava location, accounting for 18 out of 20 cases, while 2 cases were at the iliocava location. 13 out of 20 patients received a bifurcated stent-graft, while 7 out of 20 patients received an AUI (aorto-uni-iliac) stent-graft with bypass. In Type II endoleaks, the criminal vessels primarily include the IMA (inferior mesenteric artery), LA (lumbar artery), HA (hypogastric artery), and RA (renal artery). Type I endoleaks are primarily treated by extension with branch stents or cuffs, while Type II and III endoleaks are mainly managed conservatively (9/15, 60%). In addition, for Type II endoleaks, treatment options include IVC stenting, closing the fistula with occluder, and embolization or ligation of the criminal vessels. According to the follow-up results, most cases were resolved, with only 2 cases of conservatively treated patients remaining persistent endoleaks.

**Table 1:** Management of endoleaks after endovascular stent-graft of AAA with ACF.

	Type 1		Type 2	Type 3
	1A	1B		
Case number, *n*	4	1	14	1
Sex: male/female, *n*	4/0	1/0	13/1	1/0
Age, mean (yrs)	76.8 ± 1.6	80	66.6 ± 11.9	64
Fistula site, *n* (%)				
AC	4 (100%)	1 (100%)	12 (85.7%)	1 (100%)
IC	0	0	2 (14.3%)	0
Surgical methods, *n* (%)				
Bifurcated stent-graft	3 (75%)	1 (100%)	9 (64.3%)	0
AUI stent-graft + bypass	1 (25%)	0	5 (35.7%)	1 (100%)
Criminal vessels, *n* (%)				
IMA	–	–	2 (14.3%)	–
LA	–	–	4 (28.6%)	–
HA	–	–	1 64.3%)	–
RA	–	–	1 (35.7%)	–
Treatment methods, *n* (%)				
Conservation	–	–	8 (57.1%)	1
IVC Stent or occluder	–	–	4 (28.6%)	–
Embolization or ligation	–	–	2 (14.3%)	–
CUFF or extension	4 (100%)	1 (100%)	–	–
Follow-up, mean (mo)	10.5 ± 2.6	1	13.8 ± 9.7	6

AAA, abdominal aortic aneurysm; ACF, aortocaval fistula; AC, aortocava; IC, iliocava; AUI, aorto-uni-iliac; IMA, inferior mesenteric artery; LA, lumbar artery; HA, hypogastric artery; RA, renal artery; IVC, inferior vena cava.

## 4 Conclusion

In conclusion, ACF is one of the most devastating and rare complications of AAA. Early diagnosis enables significant clinical improvement because of the early reversal of hepatic, renal, and cardiovascular complications. Endovascular repair is an effective option to traditional open surgical repair, especially in patients with multiple comorbidities. However, the perioperative endoleak is the most common complication of endovascular repair, especially in patients with a great angle at the proximal neck or a short proximal neck. A conservative management may be preferable for low-flow type II endoleaks, whereas high-flow type I, type II, and type III endoleaks need to be repaired by surgical or endovascular methods.

## Data Availability

The original contributions presented in the study are included in the article/Supplementary Material, further inquiries can be directed to the corresponding authors.
